# Influence of Dentin Desensitizer Pretreatment on the Shear Bond Strength of Composite Resin Using Three Adhesive Systems: An In Vitro Study

**DOI:** 10.7759/cureus.114252

**Published:** 2026-08-10

**Authors:** M Jhansi, KS Chandrababu, Sunilkumar C, Vamsee Krishna N, S. Sunil Kumar, Ramisetty Bharathisuma

**Affiliations:** 1 Department of Conservative Dentistry and Endodontics, Chadalawada Krishna Srinivasa (CKS) Teja Institute of Dental Sciences and Research, Tirupati, IND

**Keywords:** adhesive dentistry, composite resin, dentin bonding agents, dentin desensitizer, shear bond strength

## Abstract

Background: Dentin desensitizers are used to reduce postoperative sensitivity but may influence the bonding performance of adhesive systems. The effect of dentin desensitizers on different generations of dentin bonding agents requires further evaluation.

Aim: To compare and evaluate the shear bond strength of composite resin using fifth-, seventh-, and eighth-generation bonding agents with and without the application of a dentin desensitizer.

Materials and methods: Sixty extracted human permanent molars were randomly divided into six groups (n = 10). Composite restorations were performed using three generations of bonding agents with or without Prevest Shield Activ (Prevest DenPro, Jammu, India) dentin desensitizer. Shear bond strength was evaluated using a Universal Testing Machine (Instron®, Norwood, MA, USA). Data were analyzed using one-way analysis of variance (ANOVA) and Tukey's post hoc test for multiple group comparisons. Statistical analysis was set at p<0.05.

Results: The fifth-generation adhesive without desensitizer demonstrated the highest shear bond strength (24.75 ± 1.15 MPa), whereas the seventh-generation adhesive with desensitizer showed the lowest (14.52 ± 0.61 MPa). Significant intergroup differences were observed (F = 167.516, p<0.001). Application of the dentin desensitizer reduced bond strength in all adhesive systems.

Conclusion: The fifth-generation adhesive exhibited superior shear bond strength. Pretreatment with dentin desensitizer reduced bond strength in all groups, with the greatest reduction observed in the seventh-generation adhesive. Careful selection of adhesive systems is recommended when dentin desensitizers are used clinically.

## Introduction

Resin-based composite restorations have become the treatment of choice for the restoration of both anterior and posterior teeth because of their superior esthetics, conservative tooth preparation, and continuous improvements in adhesive technology. The longevity and clinical success of these restorations depend largely on the quality of adhesion achieved between the restorative material and the tooth substrate. Reliable bonding not only enhances retention but also minimizes marginal leakage, secondary caries, postoperative sensitivity, and restoration failure [1,2].

Adhesive dentistry has undergone remarkable evolution since Buonocore first introduced the acid-etch technique in 1955, which revolutionized restorative dentistry by enabling micromechanical retention of resin materials to enamel. Subsequent advancements led to the development of dentin bonding systems capable of forming a stable hybrid layer through infiltration of resin monomers into demineralized dentin. The continuous refinement of adhesive formulations has resulted in simplified clinical protocols while aiming to improve bond durability and long-term clinical performance [3,4].

Bonding to dentin remains considerably more challenging than bonding to enamel because of its heterogeneous composition, high organic content, tubular architecture, intrinsic moisture, and outward dentinal fluid movement. These characteristics make dentin a dynamic substrate that is highly technique-sensitive and susceptible to degradation of the adhesive interface over time. Therefore, achieving durable dentin adhesion continues to be a major focus of restorative research [5,6].

Dentin bonding agents are generally classified according to their application strategy and generation. Fifth-generation adhesives employ a two-step etch-and-rinse approach, in which a combined primer and adhesive is applied after phosphoric acid conditioning. These systems have consistently demonstrated excellent bond strength because complete smear layer removal facilitates deeper resin infiltration and hybrid layer formation [7].

Seventh-generation adhesives were introduced to simplify clinical procedures by combining etchant, primer, and adhesive into a single solution. Although these self-etch systems reduce chairside time and technique sensitivity, their relatively mild etching capability and increased hydrophilicity may result in thinner hybrid layers, reduced resin tag formation, and greater susceptibility to hydrolytic degradation [8].

More recently, eighth-generation or universal adhesives have incorporated functional monomers such as 10-methacryloyloxydecyl dihydrogen phosphate (10-MDP) together with nanofillers to improve chemical interaction with hydroxyapatite, enhance bond durability, and provide versatility through self-etch, selective-etch, or etch-and-rinse application modes. These adhesives are designed to achieve reliable adhesion while simplifying clinical procedures and improving long-term stability [9,10].

Despite improvements in adhesive systems, postoperative dentin hypersensitivity remains a common clinical concern following composite restorations. According to Brännström's hydrodynamic theory, external stimuli produce movement of dentinal fluid within exposed dentinal tubules, stimulating pulpal nerve endings and producing pain. Consequently, dentin desensitizing agents are frequently applied before restorative procedures to reduce dentin permeability and improve patient comfort [11].

Among the available desensitizing agents, glutaraldehyde- and 2-hydroxyethyl methacrylate (HEMA)-containing formulations, bioactive calcium phosphate materials, bioactive glass, and potassium nitrate-based products have shown clinical effectiveness in occluding dentinal tubules. Shield Activ (Prevest DenPro, Jammu, India), a recently introduced desensitizer containing HEMA, sodium fluoride, potassium nitrate, and ethanol, reduces dentin hypersensitivity by physically sealing dentinal tubules while promoting improved wettability of the dentin surface [12].

Although dentin desensitizers effectively reduce hypersensitivity, concerns remain regarding their influence on adhesive performance. Tubular occlusion may interfere with resin monomer penetration, alter hybrid layer formation, and subsequently affect bond strength. Previous investigations have reported conflicting findings, with some studies demonstrating no significant effect, whereas others have observed either improvement or reduction in bond strength depending on the adhesive system employed [13,14].

Furthermore, limited evidence is available regarding the interaction of contemporary eighth-generation universal adhesives with dentin desensitizers in comparison with conventional fifth- and seventh-generation systems. Understanding these interactions is clinically important for selecting adhesive protocols that simultaneously minimize postoperative sensitivity and maximize restoration longevity.

Therefore, the present in vitro study was undertaken to compare the shear bond strength of composite restorations using fifth-, seventh-, and eighth-generation dentin bonding agents with and without prior application of a dentin desensitizer.

The aim of the present in vitro study was to compare and evaluate the shear bond strength of composite resin bonded with fifth-, seventh-, and eighth-generation dentin bonding agents in the presence and absence of a dentin desensitizer. The study was designed to assess the individual shear bond strength of each adhesive system, determine the effect of prior application of a dentin desensitizer on adhesive performance, and compare the bonding efficacy among the three generations of dentin bonding agents to identify the adhesive protocol that provides the most favorable bond strength under the tested conditions.

## Materials and methods

This in vitro study was conducted in the Department of Conservative Dentistry and Endodontics, C.K.S. Theja Institute of Dental Sciences and Research, Tirupati (#CKS/ENDO/23-24/02), to evaluate the effect of a dentin desensitizer on the shear bond strength of composite resin using three different generations of dentin bonding agents. A total of 60 freshly extracted, non-carious human permanent molars were selected for the study. Teeth exhibiting intact coronal structure without caries, restorations, cracks, fluorosis, developmental defects, or non-carious cervical lesions were included, whereas fractured, restored, non-vital, or structurally compromised teeth were excluded. Following extraction, all specimens were cleaned of debris and calculus using hand scalers and stored in distilled water at 37°C for 24 hours until use.

The teeth were decoronated at the cemento-enamel junction using a diamond disc under continuous water irrigation. The coronal portions were embedded in self-cure acrylic resin blocks with the dentin surfaces oriented parallel to the horizontal plane. Standardized flat dentin surfaces were prepared using a high-speed handpiece with a cylindrical diamond bur under water coolant, followed by polishing with silicon carbide abrasive paper to obtain a smooth bonding surface.

The specimens were randomly divided into six experimental groups (n = 10) according to the bonding agent used and the application of dentin desensitizer. Group I received a fifth-generation etch-and-rinse adhesive (One Coat Bond SL, Coltene AG, Altstätten, Switzerland); Group II received a seventh-generation self-etch adhesive (BeautiBond Shofu Inc., Kyoto, Japan); Group III received an eighth-generation universal adhesive (Tetric N-Bond Universal Ivoclar Vivadent AG, Schaan, Liechtenstein); Group IV received Prevest Shield Activ (Prevest Denpro Ltd., Jammu, India) dentin desensitizer followed by the fifth-generation adhesive, a self-etch adhesive system; Group V received dentin desensitizer followed by the seventh-generation adhesive; and Group VI received dentin desensitizer followed by the eighth-generation adhesive.

For Groups I to III, the dentin surfaces were etched with 37% phosphoric acid for 15 seconds, rinsed for 20 seconds, gently blot-dried to maintain moist dentin, and the respective adhesive systems were applied according to the manufacturers' instructions and light cured. In Groups IV to VI, the dentin desensitizer (Prevest Shield Activ, Prevest Denpro Ltd., Jammu, India) was first applied using a microbrush for 10 seconds, allowed to remain undisturbed for 20 seconds, gently air-dried, and subsequently the respective bonding agents were applied and light cured.

Composite resin (Tetric N-Ceram, Ivoclar Vivadent AG, Schaan, Liechtenstein, shade A2) was placed using a standardized cylindrical mould with an internal diameter of 4 mm and a height of 4 mm, in 2-mm increments, with each increment light cured for 40 seconds using an LED curing unit. Following restoration, all specimens were stored in distilled water at 37°C until mechanical testing.

**Figure 1:**
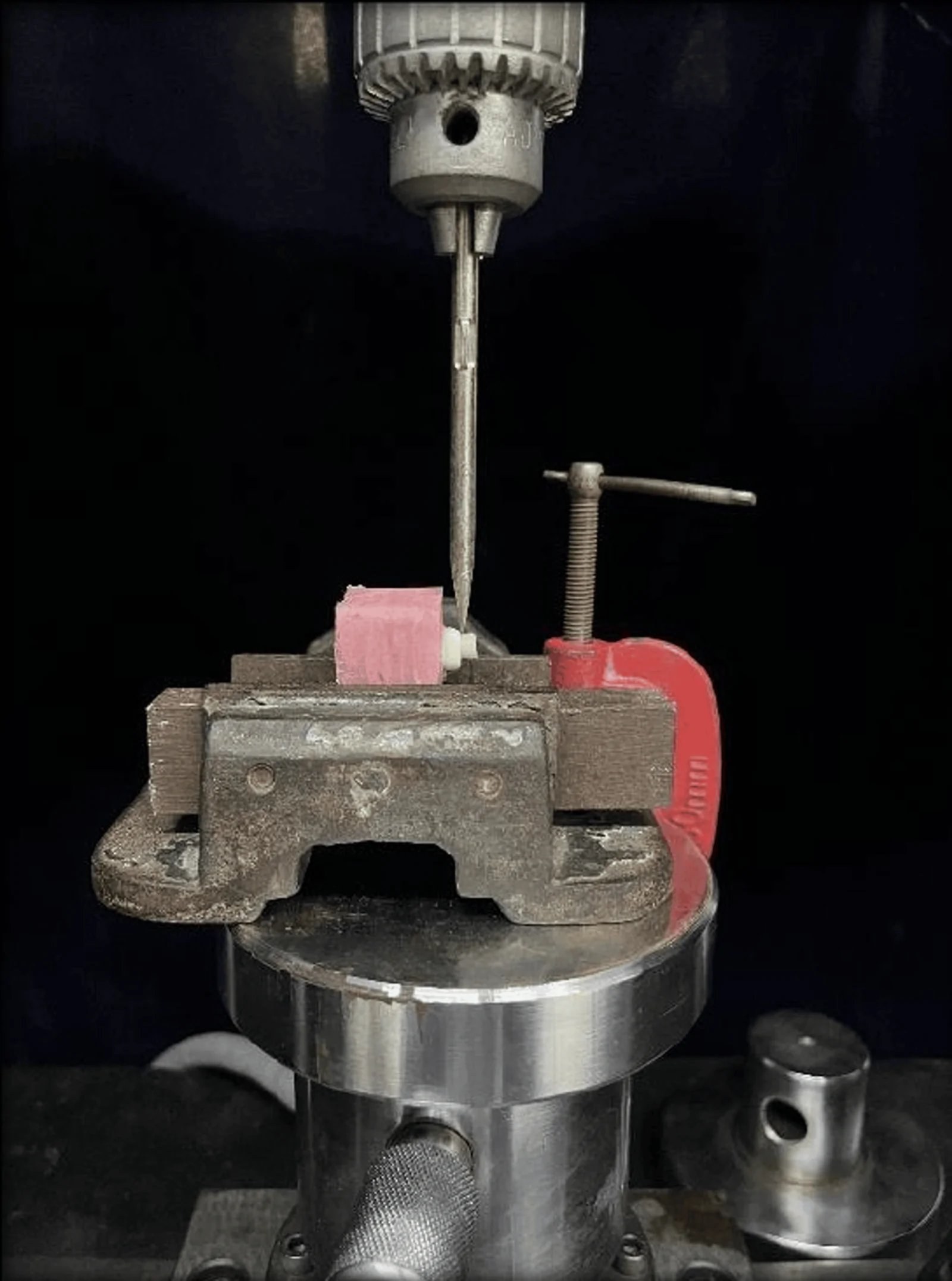
Shear bond strength evaluated using Universal Testing Machine.

The shear bond strength of all specimens was evaluated using a Universal Testing Machine (Instron®, Norwood, MA, USA) by applying a shear load at the composite-tooth interface until bond failure occurred (Figure 1). The maximum load at failure was recorded and converted to shear bond strength values expressed in megapascals (MPa). Statistical analysis was performed using one-way analysis of variance (ANOVA) to compare the mean shear bond strength among the six groups, followed by Tukey's post hoc test for multiple pairwise comparisons. A p-value < 0.05 was considered statistically significant.

Statistical analysis was performed using SPSS software (version 28.0; IBM Corp., Armonk, NY, USA). Data were assessed for normality prior to analysis. Continuous variables were expressed as mean ± standard deviation (SD). Differences in shear bond strength among the six experimental groups were analyzed using one-way analysis of variance (ANOVA). Tukey's post hoc test was performed for multiple significant differences observed. A p-value <0.05 was considered statistically significant.

## Results

**Figure 2:**
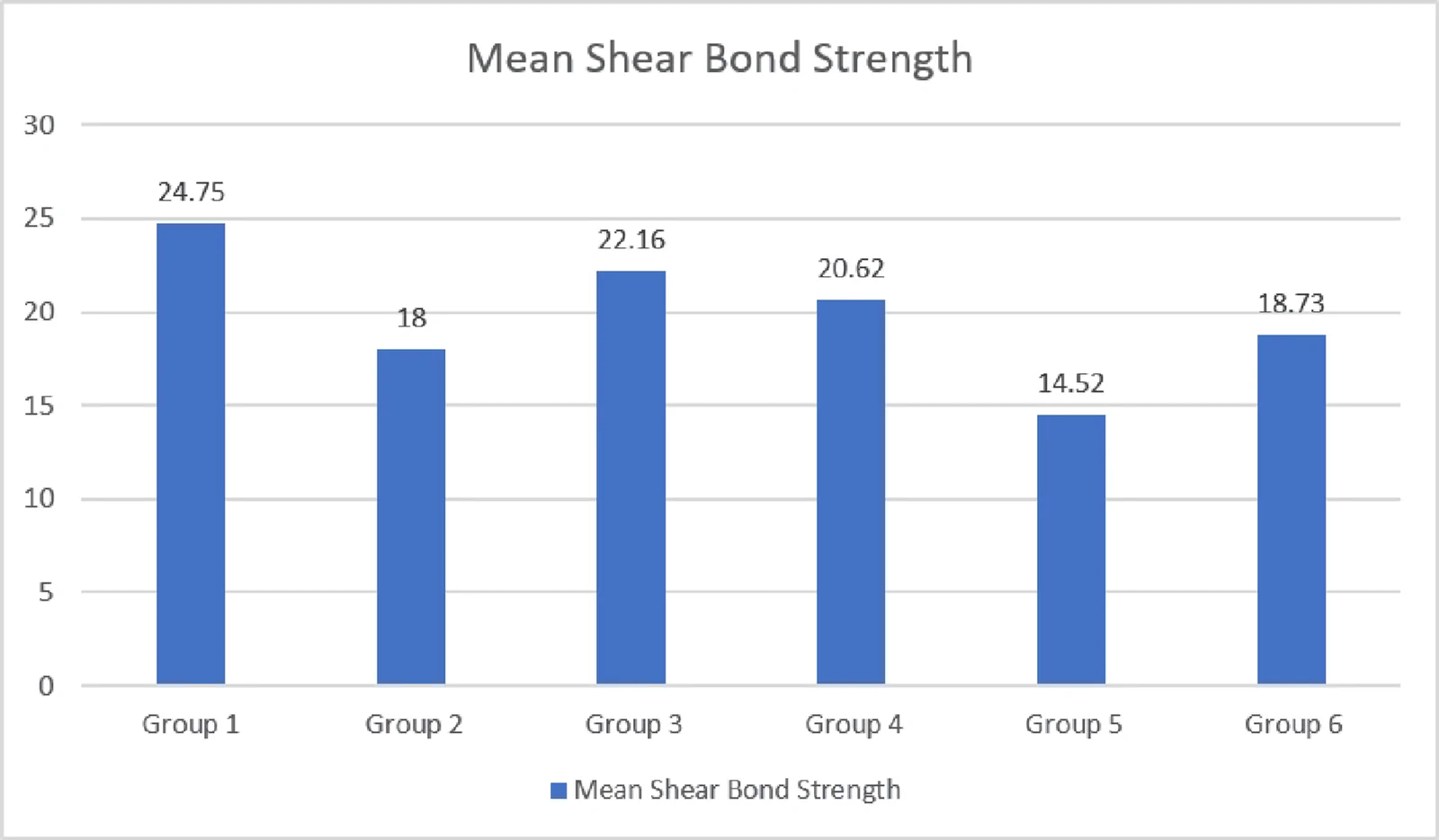
Bar graph showing the mean shear bond strength values of Groups 1, 2, 3, 4, 5, and 6.

A total of 60 specimens were included in the study and equally distributed into six experimental groups (n = 10). The mean shear bond strength values differed significantly among the groups (Figure 2). The highest mean shear bond strength was observed in Group I (fifth-generation bonding agent without dentin desensitizer) (24.75 ± 1.15 MPa), followed by Group III (eighth-generation bonding agent without dentin desensitizer) (22.16 ± 0.90 MPa), Group IV (fifth-generation bonding agent with dentin desensitizer) (20.62 ± 0.80 MPa), Group VI (eighth-generation bonding agent with dentin desensitizer) (18.73 ± 0.73 MPa), and Group II (seventh-generation bonding agent without dentin desensitizer) (18.00 ± 0.91 MPa). The lowest mean shear bond strength was recorded in Group V (seventh-generation bonding agent with dentin desensitizer) (14.52 ± 0.61 MPa) (Table 1).

**Table 1:** Comparison of shear bond strength among the experimental groups.

Group	Experimental group	n	Mean ± SD (MPa)
Group I	Fifth-generation adhesive	10	24.75 ± 1.15
Group II	Seventh-generation adhesive	10	18.00 ± 0.91
Group III	Eighth-generation adhesive	10	22.16 ± 0.90
Group IV	Fifth-generation adhesive + dentin desensitizer	10	20.62 ± 0.80
Group V	Seventh-generation adhesive + dentin desensitizer	10	14.52 ± 0.61
Group VI	Eighth-generation adhesive + dentin desensitizer	10	18.73 ± 0.73

One-way analysis of variance (ANOVA) demonstrated a statistically highly significant difference in shear bond strength among the six experimental groups (F = 167.516, p < 0.001), indicating that both the type of bonding agent and the application of dentin desensitizer significantly influenced adhesive performance (Table 2).

**Table 2:** Comparison of shear bond strength using one-way ANOVA. ANOVA: analysis of variance. *Statistically significant.

Parameter	Value
Between-group sum of squares	683.92
Within-group sum of squares	44.07
Degrees of freedom (between/within)	5/54
F-value	167.516
p-value	<0.001*

Post hoc analysis using Tukey's multiple comparison test revealed statistically significant differences between almost all group pairs (p < 0.05). However, the comparison between Group II (seventh-generation adhesive without desensitizer) and Group VI (eighth-generation adhesive with desensitizer) was not statistically significant (p = 0.424) (Table 3).

**Table 3:** Multiple comparison of shear bond strength among experimental groups (Tukey's post hoc test) NS: not significant. *Statistically significant.

Comparison	Mean difference (MPa)	p-value
Group I vs II	6.75	<0.001*
Group I vs III	2.59	<0.001*
Group I vs IV	4.13	<0.001*
Group I vs V	10.23	<0.001*
Group I vs VI	6.02	<0.001*
Group II vs III	-4.16	<0.001*
Group II vs IV	-2.62	<0.001*
Group II vs V	3.48	<0.001*
Group II vs VI	-0.73	0.424 (NS)
Group III vs IV	1.54	0.003*
Group III vs V	7.64	<0.001*
Group III vs VI	3.43	<0.001*
Group IV vs V	6.10	<0.001*
Group IV vs VI	1.89	<0.001*
Group V vs VI	-4.21	<0.001*

Application of the dentin desensitizer resulted in a reduction in shear bond strength for all three adhesive systems. The fifth-generation adhesive exhibited the highest bond strength irrespective of desensitizer application, whereas the seventh-generation adhesive demonstrated the greatest reduction following pretreatment with the dentin desensitizer. The eighth-generation universal adhesive showed intermediate bond strength, performing better than the seventh-generation adhesive but lower than the fifth-generation adhesive under both conditions.

## Discussion

The present in vitro study evaluated the effect of a dentin desensitizer on the shear bond strength of composite resin bonded with fifth-, seventh-, and eighth-generation dentin bonding agents. Shear bond strength is a widely accepted laboratory parameter for assessing the adhesive performance and durability of restorative materials, as it reflects the ability of the adhesive interface to withstand functional stresses [15].

The findings of the present study demonstrated significant differences in shear bond strength among the experimental groups. The fifth-generation adhesive without dentin desensitizer exhibited the highest bond strength, whereas the seventh-generation adhesive with dentin desensitizer showed the lowest values. These results indicate that both the type of adhesive system and the application of a dentin desensitizer significantly influence bonding effectiveness.

The superior performance of the fifth-generation adhesive can be attributed to its etch-and-rinse mechanism, which completely removes the smear layer and facilitates deeper resin infiltration into the demineralized dentin, resulting in a thicker hybrid layer and stronger micromechanical retention. Similar findings were reported by Hegde et al. [16], who observed significantly higher shear bond strength with total-etch adhesives than with self-etch systems. Likewise, Ashhar et al. [17] demonstrated that fifth-generation adhesives produced superior bond strength because of effective hybrid layer formation, while Gangurde et al. [18] also reported excellent bonding performance with fifth-generation dentin bonding agents.

The eighth-generation universal adhesive demonstrated higher bond strength than the seventh-generation adhesive but lower values than the fifth-generation adhesive. This may be attributed to the presence of functional monomers such as 10-MDP, which provide both micromechanical and chemical bonding with hydroxyapatite, thereby improving adhesive stability. Similar observations were made by Chauhan et al. [19], Khudhur et al. [20], and Basheer et al. [21], who reported superior bond strength of eighth-generation adhesives compared with simplified self-etch systems.

In contrast, the seventh-generation adhesive exhibited comparatively lower bond strength. The all-in-one formulation, increased hydrophilicity, and relatively mild etching ability may limit resin infiltration and hybrid layer formation. These findings are in agreement with Shafigh et al. [22] and Fulari et al. [23], who also observed lower bond strength with seventh-generation adhesive systems.

An important finding of the present study was that application of the dentin desensitizer reduced shear bond strength in all adhesive systems. This reduction may be explained by occlusion of dentinal tubules, which restricts adhesive penetration and compromises hybrid layer formation. Similar findings were reported [23], indicating that dentin desensitizers influenced bond strength depending on the adhesive strategy used. Likewise, Ashraf et al. [24] reported that desensitizer pretreatment adversely affected the bonding performance of self-etch adhesives. A systematic review [24] also concluded that dentin desensitizers can alter adhesive performance depending on their composition and the adhesive system employed.

Despite the reduction in bond strength after desensitizer application, the fifth-generation adhesive maintained the highest bond strength among the desensitizer-treated groups. This finding is comparable with that of Ravikumar et al. [25], who reported that etch-and-rinse adhesives are less affected by dentin desensitizers because phosphoric acid etching improves resin penetration despite partial tubular occlusion.

The greatest reduction in bond strength was observed with the seventh-generation adhesive combined with the dentin desensitizer. The combined effect of dentinal tubule occlusion and the limited etching capacity of simplified self-etch adhesives probably reduced resin infiltration and adhesive polymerization. Similar observations were reported by Shahidi et al. [26] and Jain et al. [27], who concluded that the influence of desensitizers varies according to the adhesive generation and bonding strategy.

Overall, the findings of the present study indicate that both adhesive generation and dentin desensitizer application significantly influence shear bond strength. Among the adhesive systems evaluated, the fifth-generation etch-and-rinse adhesive demonstrated the most favorable bonding performance, whereas the seventh-generation self-etch adhesive was the most adversely affected by dentin desensitizer application.

From a clinical perspective, these findings suggest that clinicians should carefully consider the compatibility between dentin desensitizers and adhesive systems before restorative procedures; inappropriate combinations may compromise bond durability and restoration longevity. Nevertheless, long-term in vitro studies incorporating artificial aging and randomized clinical trials are required to validate these findings under clinical conditions.

Limitations

The present in vitro study was conducted under in vitro conditions and therefore may not completely replicate the complex oral environment, where factors such as saliva, pulpal pressure, temperature fluctuations, masticatory forces, and enzymatic activity may influence adhesive performance. The specimens were stored in distilled water at 37°C for 24 hours, and no artificial aging procedures, such as thermocycling or cyclic loading, were performed. In addition, failure mode analysis of fractured specimens was not carried out, limiting the interpretation of the mode of bond failure. Furthermore, only one dentin desensitizer and three adhesive systems were evaluated.

Future research

Future studies should evaluate the long-term durability of the adhesive interface using thermocycling, cyclic loading, and extended water storage. Evaluation of different commercially available dentin desensitizers, an additional universal adhesive system, and failure mode analysis using electron microscopy would provide a better understanding of the bonding mechanism. Well-designed randomized clinical trials are also required to determine whether the present in vitro findings translate into improved clinical performance and restoration longevity.

## Conclusions

Within the limitations of this in vitro study, it can be concluded that the shear bond strength of composite resin is significantly influenced by both the generation of the dentin bonding agent and the application of a dentin desensitizer. Among the adhesive systems evaluated, the fifth-generation etch-and-rinse adhesive demonstrated the highest shear bond strength, indicating superior bonding performance. The eighth-generation universal adhesive exhibited better bond strength than the seventh-generation adhesive but remained inferior to the fifth-generation adhesive. Application of the dentin desensitizer resulted in a reduction in shear bond strength in all adhesive systems, with the greatest reduction observed in the seventh-generation self-etch adhesive. Despite this reduction, the fifth-generation adhesive maintained comparatively higher bond strength following desensitizer application. These findings suggest that when the use of a dentin desensitizer is clinically indicated, selection of an appropriate adhesive system is essential to achieve optimal bond strength and enhance the long-term success of composite restorations. Further long-term in vivo studies are recommended to validate these findings under clinical conditions.
